# Effects of DORA Daridorexant on insomnia disorder in patients with comorbid unipolar and bipolar depression: data from a naturalistic longitudinal study

**DOI:** 10.1192/j.eurpsy.2025.567

**Published:** 2025-08-26

**Authors:** L. Anastasio, G. Alfi, G. Aquino, E. Annuzzi, G. Cerofolini, A. Coccoglioniti, M. Gambini, M. Pioltino, G. Perugi, A. Gemignani, L. Palagini

**Affiliations:** 1 Department of Neuroscience, Psychiatric Unit, Azienda Ospedaliera Universitaria Pisana (AUOP), University ofPisa, Pisa, Italy; 2 University of Pisa, Pisa, Italy

## Abstract

**Introduction:**

Insomnia is the most *common* sleep disorder, which may *favor*, precipitate, and prolong mental disturbances, including mood disorders. *The treatment of* insomnia in the context of mood disorders may contribute to *improving* their trajectories*, possibly by* improving mood symptoms via sleep regulation. Daridorexant is a new *pharmacological option for insomnia treatment,* which is a *dual orexin receptor antagonist (DORA).*

**Objectives:**

The aim of the present study was to treat consecutive patients with insomnia disorder with and without mental co-morbidities with this new therapeutic option.

**Methods:**

Ninety consecutive patients with insomnia disorder according to the DSM-5-TR criteria were treated with daridorexant 50 mg. Baseline, 1 month, and 3-month evaluations were performed. Demographic and clinical data were incorporated. Insomnia severity (Insomnia Severity Index-ISI), mood symptoms (Beck Depression Inventory II-BDI-II, Young Mania Rating Scale-YMRS), suicidal ideation (Suicidal Ideation Scale-SSI), and emotional dysregulation (Difficulties in Emotion Regulation Scale-DERS) were evaluated. The evaluation of psychiatric diagnosis was conducted in accordance with the DSM-5-TR criteria (SCID-5) and the concurrent use of pharmacological therapy was taken into account.

**Results:**

The final sample included 80 patients (N° 40, 50.0% females, mean age 60 ± 13.2). Most of them (N°50 62.5%) suffered from insomnia comorbid with unipolar/bipolar depression. Repeated Anova analyses showed that ISI and DERS total score decreased across time (respectively F=63.42, p<0.001, F=41.12, p<0.001). Similarly depressive and mixed symptoms, suicidal ideation and anxiety symptoms significantly improved over time and after 3 months of treatment (respectively F=62.45, p<0.001, F=31.48, p<0.001, F=41.14, p<0.001, F=21.44, p<0.001). Analyses conducted on a subsample of patients with comorbid unipolar and bipolar disorder revealed a distinct beneficial effect on insomnia and mood symptoms. Multiple regression models demonstrated that improvement of depressive symptoms was best predicted by the improvement in emotion regulation -DERS score at T1 and T2, and the improvement in insomnia symptoms at T1. An improvement in manic symptoms-YMRS score was best predicted by an improvement in insomnia symptoms-ISI score and in emotion regulation at T1 and T2.

**Conclusions:**

The treatment of chronic insomnia with daridorexant improved insomnia symptoms in patients with and without mental comorbidities across time. It was particularly effective in patients with unipolar and bipolar disorders, where the improvement in mood symptoms was related to the improvement in insomnia across time. These data are in line with the data showing that targeting insomnia in the context of mood disorders might be useful for improving sleep and mood symptoms by regulating the sleep system.

**Disclosure of Interest:**

L. Anastasio: None Declared, G. Alfi: None Declared, G. Aquino: None Declared, E. Annuzzi: None Declared, G. Cerofolini: None Declared, A. Coccoglioniti: None Declared, M. Gambini: None Declared, M. Pioltino: None Declared, G. Perugi: None Declared, A. Gemignani: None Declared, L. Palagini Consultant of: Consultancy for Laura Palagini, Bruno, Idorsia, Fidia, Pfizer, Sanofi

